# Bacterial community composition of chronic periodontitis and novel oral sampling sites for detecting disease indicators

**DOI:** 10.1186/2049-2618-2-32

**Published:** 2014-08-26

**Authors:** Vaia Galimanas, Michael William Hall, Natasha Singh, Michael David Joseph Lynch, Michael Goldberg, Howard Tenenbaum, Dennis Gerard Cvitkovitch, Josh David Neufeld, Dilani Braziunas Senadheera

**Affiliations:** 1 Dental Research Institute, University of Toronto, 124 Edward Street, Toronto, ON M51G6, Canada; 2 Department of Biology, University of Waterloo, Waterloo, Ontario N2L 3G1, Canada

**Keywords:** Oral microbiome, 16S rRNA gene, Chronic periodontitis, Tongue, Supragingival, Subgingival, Plaque, Bacterial community

## Abstract

**Background:**

Periodontitis is an infectious and inflammatory disease of polymicrobial etiology that can lead to the destruction of bones and tissues that support the teeth. The management of chronic periodontitis (CP) relies heavily on elimination or at least control of known pathogenic consortia associated with the disease. Until now, microbial plaque obtained from the subgingival (SubG) sites has been the primary focus for bacterial community analysis using deep sequencing. In addition to the use of SubG plaque, here, we investigated whether plaque obtained from supragingival (SupG) and tongue dorsum sites can serve as alternatives for monitoring CP-associated bacterial biomarkers.

**Results:**

Using SubG, SupG, and tongue plaque DNA from 11 healthy and 13 diseased subjects, we sequenced V3 regions (approximately 200 bases) of the 16S rRNA gene using Illumina sequencing. After quality filtering, approximately 4.1 million sequences were collapsed into operational taxonomic units (OTUs; sequence identity cutoff of >97%) that were classified to a total of 19 phyla spanning 114 genera. Bacterial community diversity and overall composition was not affected by health or disease, and multiresponse permutation procedure (MRPP) on Bray-Curtis distance measures only supported weakly distinct bacterial communities in SubG and tongue plaque depending on health or disease status (*P* < 0.05). Nonetheless, in SubG and tongue sites, the relative abundance of *Firmicutes* was increased significantly from health to disease and members of *Synergistetes* were found in higher abundance across all sites in disease. Taxa indicative of CP were identified in all three locations (for example, *Treponema denticola*, *Porphyromonas gingivalis*, *Synergistes* oral taxa 362 and 363).

**Conclusions:**

For the first time, this study demonstrates that SupG and tongue dorsum plaque can serve as alternative sources for detecting and enumerating known and novel bacterial biomarkers of CP. This finding is clinically important because, in contrast with SubG sampling that requires trained professionals, obtaining plaque from SupG and tongue sites is convenient and minimally-invasive and offers a novel means to track CP-biomarker organisms during treatment outcome monitoring.

## Background

Bacterial communities that colonize the human body are intimately linked with host physiology, immunity, metabolism, and nutrition [1-3]. Of colonization sites, the mouth accommodates a bacterial consortium with high taxonomic richness and phylogenetic diversity [4-6]. The microbial composition of the plaque biofilm has a critical role in oral health. Disruption of plaque homeostasis can stimulate tissue destruction and inflammation, leading to infections such as dental caries, gingivitis, and periodontitis [4,7]. A comprehensive and systematic profiling of the oral biofilm is necessary, not only to understand microbial associations with localized infections, but also because the oral microbiome has long been known as a reservoir for infections at other body sites [8,9]. Bacterial species normally found in the oral cavity have been associated with distal infections in the lungs, heart, brain, and liver, either reflecting their involvement in opportunistic infections as a result of systemic changes in the body, or perhaps suggesting a causative link [8,10-13]. Therefore, the plaque consortia can be a mirror and possibly even a monitor of oral *and* non-oral health and disease.

Periodontitis is a chronic inflammatory disease affecting tooth-supporting structures including the alveolar bone, connective tissue attachment, and gingiva [14,15]. Although several forms of periodontal diseases have been recognized, the predominant category is chronic periodontitis (CP), which remains a primary cause of tooth loss in adults worldwide [16]. In addition to its large socio-economic burden [17], CP is associated with considerable morbidity in terms of pain, uncomfortable chewing, oral malodor, and tooth migration. Although the pathogenesis of periodontitis is multifactorial and includes genetic and epigenetic factors, the development of periodontitis is modulated by microbial biofilm that forms on and around teeth, eliciting an inflammatory host reaction [18-21]. The etiology of periodontitis is polymicrobial. In particular, disease progression has been linked with the proliferation of Gram-negative anaerobic species such as *Porphyromonas gingivalis*, *Treponema denticola*, and *Tannerella forsythia*[22].

Over the past two decades, culture-based, immunohistochemistry, and molecular techniques have focused largely on a small subset of CP-associated microorganisms in the gingival sulcus and thus have overlooked the impact of potentially large numbers of other oral bacterial species in the infectious process [22-24]. Although these as-yet-unidentified organisms might not always be associated intimately with the periodontium at all times, it is still likely that several of them, at one time or another, participate in the pathophysiological processes that lead to and cause the advancement of CP. Emerging evidence of subgingival (SubG) community analysis using DNA sequencing suggests that periodontal destruction is associated with many uncultivable and uncharacterized disease indicator organisms [25-27]. However, despite the growing list of periodontitis-associated bacteria, there is slow progress in the use of this information to develop more cost-effective and sensitive methods to treat, control, or prevent disease progression. It is probable that a more complete characterization of bacterial biomarkers of CP will lead to the development of new therapeutics, improved diagnostics, and alternative methods for monitoring the outcomes of treatment success.

Recently, high-throughput sequencing approaches have assessed bacterial composition in deep and shallow SubG samples derived from patients with periodontitis compared to healthy patients [25]. The results demonstrated that patients with periodontitis had more diverse combinations of species but also had all of the health-associated species, albeit at a lower frequency. These results agreed with another study that showed increasing diversity in diseased compared to healthy sites [27]. However, diversity did not increase between bleeding and non-bleeding SubG sites; an increase in total bacterial load was demonstrated in the SubG samples obtained from bleeding sites. Both of these studies noted a clear distinction between healthy and diseased SubG sites when using distance metric analysis [25,27].

In this study, we used 16S rRNA gene analyses for examining bacterial communities associated with periodontal health and CP, using plaque extracted from SubG and supragingival (SupG) as well as sites on the dorsum of the tongue. We hypothesized that consistent differences in bacterial communities of both dental and tongue plaque would exist between CP and healthy patients and that these differences would be largely due to known disease-associated taxa as well as other as-yet-uncharacterized bacteria. Another goal was to determine if SupG and tongue plaque sampling could be used as a novel and non-invasive detection method of indicator taxa for CP, instead of conventionally sampled SubG plaque. A relatively simpler alternative sampling protocol would greatly facilitate the detection of periodontitis-associated bacterial biomarkers in disease monitoring. For the first time, we provide evidence that both SupG and tongue dorsum sites may be suitable as alternative sampling sources for the detection and enumeration of selected CP-associated bacterial biomarkers. The use of tongue plaque or SupG plaque that is above the gum line to monitor oral health is convenient and circumvents the utility of specially trained personnel for the sampling procedure; in fact, samples can be provided by the patients themselves. From a clinical standpoint, this study provides important groundwork for developing a minimally invasive method to monitor CP treatment outcomes.

## Results

### Demographics, clinical attributes, and sequencing

Fifteen women and nine men in the age range of 18 to 71 years were recruited for this study. The cohort with a healthy periodontium consisted of eight women and three men in the age range of 18 to 56 years; the CP group comprised seven women and six men in the age range of 25 to 71 years (Table 1). CP was diagnosed on the basis of radiographic and clinical examination. Parameters derived from clinical evaluation included probing depth and clinical attachment loss (CAL). Bone loss on each tooth was assessed radiographically. Patients in the CP group had >30% sites with ≥5 mm of CAL and probing pocket depths of ≥6 mm, whereas the healthy group had >30% sites with ≤2 mm attachment loss and no pockets >3 mm. No patients with systemic diseases, antibiotic use, and dental cleanings within a 6-month time frame were included in the study. Differences in patient demographics and clinical data (Table 1) were assessed with a Welch’s two sample *t* test or a chi-squared test of independence. No significant differences in age, weight, gender, smoker status, or drinker status were found between health and CP. All patient and sample metadata are available in Additional file 1.

**Table 1:** Demographics and clinical parameters of participants

**Demographics, clinical parameters, and habits**	**Healthy group (*n*** **= 11)**	**Diseased group (*n*** **= 13)**
Sex	3 men; 8 women	6 men; 7 women
Age (years)	38.4 ± 4.1	46.8 ± 4.0
Weight (lbs)	146.9 ± 7.1	159.1 ± 8.2
Probing depth (mm) in > 30% of sites	≤2	≥5
Attachment loss (mm) in >30% of sites	≤1	≥3
Periodontal diagnosis^a^	Periodontal health	Chronic periodontal disease (CP)
Smokers^b^	1	6
Drinkers^c^	2	5

^a^Periodontal diagnosis based on criteria as outlined in the 1999 American Academy of Periodontology Diagnostic Guidelines; ^b^a smoker was defined as someone who had one or more cigarettes per day for >3 months; ^c^a drinker was defined as an individual who consumed ≥56 drinks per year.

For comparing bacterial community composition between participants with a healthy periodontium and CP patients, DNA isolated from plaque was subjected to PCR amplification using primers covering an approximate 200-bp segment of the V3 region of the 16S rRNA gene. DNA sequencing yielded approximately 5.8 million sequences, with a minimum of 27,187 sequences per sample (average of 57,441 ± 23,336) and between-sample analyses rarefied all sample data to 27,187 sequences. After quality filtering and PANDAseq assembly (minimum overlap of 60 nucleotides), approximately 4.1 million sequences remained. These sequences were collapsed into operational taxonomic units (OTUs) based on a pairwise sequence identity cutoff of 97%. Of the total sequences, 49,327 formed singletons and 6,160 OTUs did not classify to the phylum level (representing 66,466 sequences, approximately 0.02% of the usable sequences). OTUs were classified to a total of 19 phyla, 114 genera, and 302 species.

### Advanced periodontal disease is not correlated with changes in α-diversity

**Figure 1:**
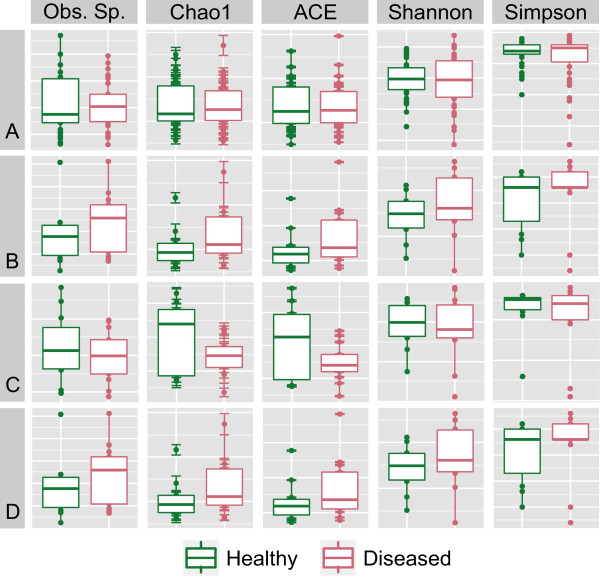
**Alpha diversity calculations for healthy (red) and diseased (blue) samples.** No significant differences between healthy and diseased samples were observed for various α-diversity measures. Calculations and plots were generated using the Phyloseq software package. **(A)** All samples (SubG, SupG, and Tongue) pooled. **(B)** SubG samples. **(C)** SupG samples. **(D)** Tongue samples.

Recent studies aimed at assessing site-specific bacterial community diversity have implicated considerable changes in the taxonomic composition in health versus disease [25,27,28]. Hence, we examined whether the biofilm community composition in the CP cohort could be correlated with consistent alterations in OTU diversity. The α-diversity within SupG, SubG, and tongue sample sites was investigated using Phyloseq. Standard diversity metrics were evaluated, including observed richness, Shannon index, and Simpson index. There were no statistically significant differences in α-diversity between healthy and diseased individuals across all sampling sites and metrics (Figure 1). The average α-diversity scores for all metrics were higher in disease for SupG and SubG sites, but lower in disease for the tongue.

### Tongue harbors a unique consortium, and periodontal destruction is associated with a weakly distinct shift relative to health

**Figure 2:**
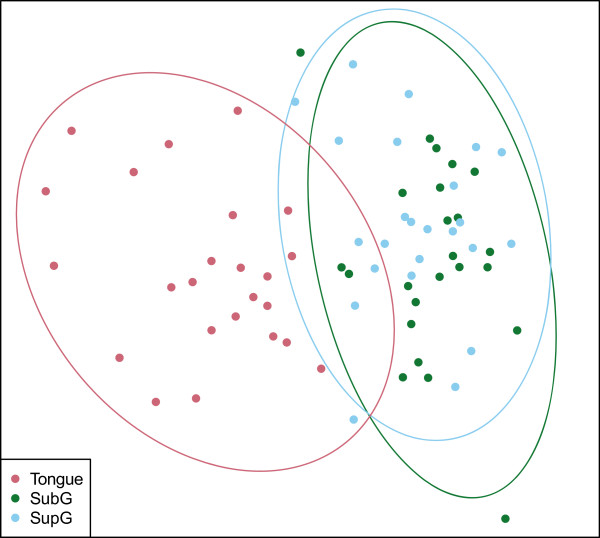
**Bray-Curtis based non-metric multidimensional scaling (NMDS) plot of all samples.** NMDS plot (stress 0.22) shows the clear clustering of tongue samples distinct from SubG and SupG samples. Ellipsoids represent a 95% confidence interval surrounding each group. MRPP analysis concluded that the members of the tongue and SubG/SupG groups were more dissimilar than expected by chance (*A* = 0.06, *T* = -22.7, *P* < 0.001).

**Figure 3:**
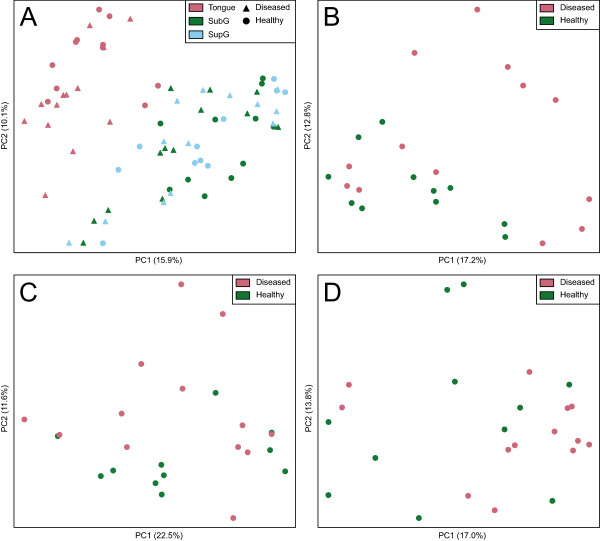
**Principal coordinate analysis (PCoA) plot with Bray-Curtis dissimilarity.** Results revealed that tongue samples clustered separately from tooth samples (SubG and SupG), suggesting that the tongue community is relatively unique from that in SupG and SubG sites. No obvious clustering was apparent within or between SupG and SubG samples. **(A)** Bray-Curtis PCoA ordination of pooled SupG, SubG, and tongue samples. **(B)** Ordination of SubG samples. Healthy patient samples are shown in green, diseased patient samples in pale red. **(C)** Ordination of SupG samples. **(D)** Ordination of tongue samples.

To assess structural similarities in the biofilm communities among SupG, SubG, and tongue sample sites in healthy and diseased patients, a multiresponse permutation procedure (MRPP) analysis based on a Bray-Curtis distance matrix was used. To visualize the results, a non-metric multidimensional scaling (NMDS) plot (Figure 2) and a principal coordinates analysis (PCoA) plot were generated (Figure 3), both based on Bray-Curtis distances. Bray-Curtis provides a measure of community composition differences between samples based on OTU counts, regardless of taxonomic assignment. Ordinations based on this metric demonstrated a clear separation of tongue samples from tooth sample locations, indicating that the bacterial community on the tongue is distinct from those in SupG and SubG sites (*A* = 0.06, *T* = -22.7, *P* < 0.001). Alternatively, there was no obvious separation between SupG and SubG samples. Similar results were shown using a PCoA plot based on UniFrac distances (plot available in Additional file 2).

Further investigation was carried out to determine whether or not there are distinct bacterial compositions in SupG, SubG, and tongue sites depending on the health status of the periodontium. For each site, a PCoA ordination and MRPP analysis, based on Bray-Curtis distances, was performed. The PCoA ordination did not reveal strong grouping of health and disease on the primary axes (Figure 3). In addition, no clear separation of health and disease samples was seen in the UniFrac-based PCoA plots (see Additional file 2). MRPP analysis of the distance matrix suggested very weak differences between health and disease samples for the combined, SubG, and tongue sites (*P* < 0.05; full MRPP results available in Additional file 3), although not for the SupG site.

### Relative abundance of distinct taxa is affected by periodontal destruction in oral plaque communities

**Figure 4:**
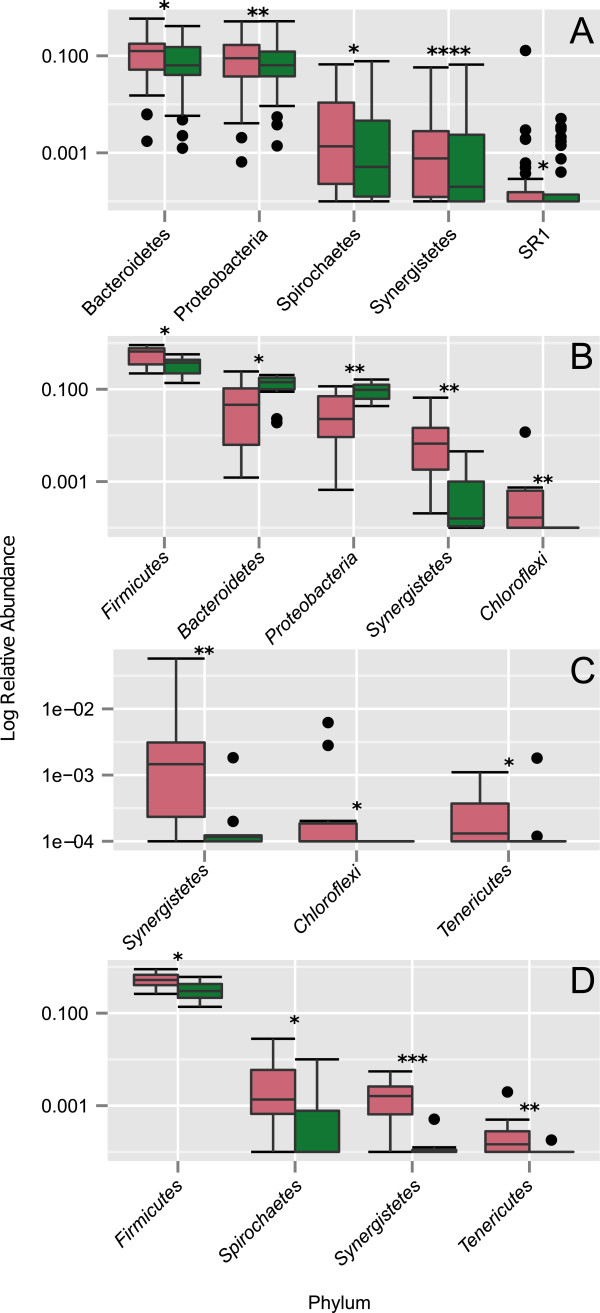
**Health and disease associated phyla, by relative abundance.** The top five most abundant phyla with significant differences between health and disease are shown. Significance was assessed with a Mann-Whitney test (*P* < 0.05). Disease abundances are shown in pale red, and health abundances in green. **(A)** All samples (SubG, SupG, and tongue). **(B)** SubG samples. **(C)** SupG samples. **(D)** Tongue samples. *0.01 < *P <* 0.05; **0.001 < *P* < 0.01; ***P < 0.001; ****P < 0.0001.

Using CP and healthy samples, the relative abundance of taxa were compared to determine whether periodontal destruction was correlated with substantial changes in the abundance of specific bacterial taxa. The abundances of each taxonomic group were assessed for significant differences between health and disease with a Mann-Whitney test. The most abundant taxa in CP-associated biofilms belonged to the phyla *Bacteroidetes* and *Proteobacteria*; the most significant difference occurred in the phylum *Synergistetes* (Figure 4). To define an association with SupG, SubG, and tongue locations, the relative abundance of taxa by sample site was also examined. In SubG and tongue samples, the relative abundance of *Firmicutes* was significantly increased under CP versus health (Figures 4B, D). Despite being associated with CP subjects in the sample site combined analysis, *Bacteroidetes* and *Proteobacteria* were found to be associated with healthy subjects in SubG samples (Figure 4B). Across all sites, the *Synergistetes* phylum was found in higher abundance in patients with CP as compared to healthy subjects (Figure 4).

Closer examination of CP-associated abundance profiles at a higher taxonomic resolution revealed many highly abundant genera in the SubG to be significantly increased in health, including *Corynebacterium*, *Capnocytophaga*, *Campylobacter*, *Neisseria*, and *Kingella* (shown in Additional file 4, panel A). On the other hand, the tongue harbored many highly abundant disease-associated genera such as *Treponema*, *Synergistes*, and *Clostridiales* (shown in Additional file 4, panel C). Examining results at the species level highlighted classic periodontal disease-associated OTUs in each of the three sites. These included *T. forsythia*, *Filifactor alocis*, and *Porphyromonas endodontalis* (see Additional file 5). In particular, *F. alocis* was found in all three sites in high abundance and significantly higher proportions in disease. Health-associated taxa included unclassified *Fusobacteria* and *Corynebacterium matruchotii* in the SubG site, *Granulicatella adiacens* and *Selenomonas artemidis* in SupG, and *S. artemidis* and unclassified *Porphyromonas* in tongue samples (significantly elevated species listed in Additional file 6).

### Periodontal destruction and periodontal health are associated with indicator organisms

**Figure 5:**
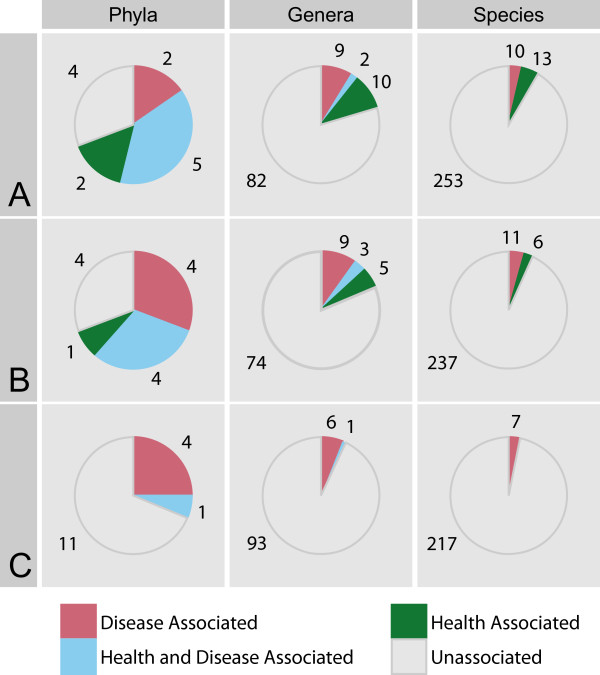
**Numbers of taxa associated with health and disease by indicator species analysis.** Dufrêne-Legendre indicator species analysis revealed OTU associations with either health or disease. The number of indicator taxa at phylum, genus, and species levels (OTU indicator value >0.5 and taxa sequence abundance ≥100) of health (green), disease (pale red), or both (light blue) are shown. Taxa that were present in the samples but not an indicator of either health or disease are shown in grey. **(A)** SubG samples. **(B)** SupG samples. **(C)** Tongue samples.

To determine if organisms present in the sample sites can serve as specific indicators of health or disease, indicator analysis of the OTUs was performed. OTUs were scored by their abundance and presence/absence in the health and disease groups. Indicator OTUs were those which were significantly more abundant and present in all samples belonging to one group and also absent or low abundance in the other group [29]. At the species level, SubG, SupG, and tongue sites of the CP cohort were associated with 10, 11, and seven species, respectively, whereas 13 and six were health-associated indicators for SubG and SupG sites, respectively (Figure 5). No indicators of health were found on the tongue at the specified indicator value threshold and abundance. In all sites, the vast majority of OTUs were not significant indicators of either health or disease.

**Figure 6:**
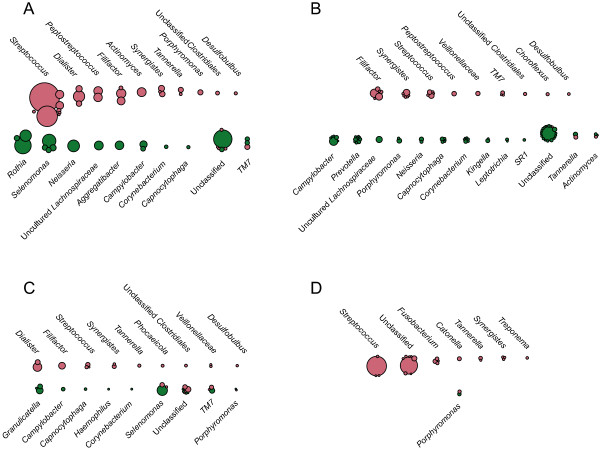
**Bubble plot of indicator OTUs associated with health and disease.** Each bubble represents an OTU identified by Dufrêne-Legendre indicator species analysis as being associated with health (green) or disease (pale red). **(A)** Pooled SupG, SubG, and tongue samples; **(B)** SubG samples; **(C)** SupG samples; and **(D)** tongue samples. Bubble area is proportional to OTU sequence abundance. Only OTUs with indicator values >0.5 and taxa with a total of ≥100 sequences are shown. Complete indicator OTU lists are available in Additional file 7.

**Figure 7:**
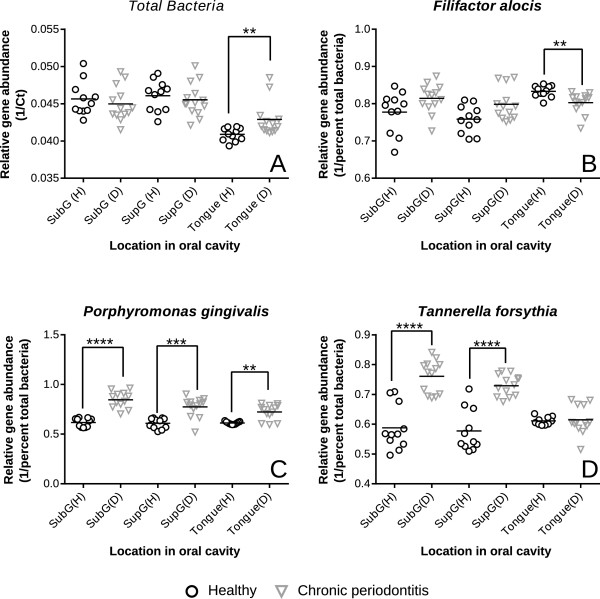
**Quantitative PCR-derived relative abundance of indicator organisms.** Data for indicators are presented as reciprocals of the mean normalized cycle threshold (Ct) values (that is, Ct_indicator species_/Ct_total bacteria_). Individual points represent average normalized expression values for each individual based on duplicate runs in both health (solid circles) and CP (inverted open triangles) for **(A)** Total bacterial load. **(B)***Filifactor alocis*, **(C)***Porphyromonas gingivalis,* and **(D)***Tannerella forsythia*. Significant differences between healthy cohorts and those with chronic periodontitis within each of the respective locations in the oral cavity is based on non-parametric Mann-Whitney Test with outliers removed using Grubbs’ Outlier Test. *0.01 < *P <* 0.05; **0.001 < *P* < 0.01; ***P < 0.001; ****P < 0.0001.

Indicator species analysis of combined data for SupG, SubG, and tongue sites showed several indicator OTUs as strong representatives of either disease or health states (Figure 6A). *Synergistes* oral taxon (OT) 363 and *Synergistes* OT 362 showed the highest indicator values for CP; *Streptococcus sanguinis*, *T. forsythia*, *F. alocis*, *Dialister invisus, Streptococcus* sp., and TM7 401H12 were also strong indicators for CP (see Additional file 6). *F. alocis* was a strong indicator of disease in SubG and SupG samples, but a relatively weak indicator of disease on the tongue (indicator value of 0.46; not shown in Figure 7). Interestingly, *T. forsythia*, as well as the unclassified *Synergistes* OT 363 and 362, were indicators of disease in this investigation (Figure 6). Health indicators included unclassified *Fusobacteriales*, TM7 7BB428, *Campylobacter rectus*, uncultured *Lachnospiraceae*, OT 100, unclassified *Bacteroidetes*, *Aggregatibacter*, *Capnocytophaga gingivalis, C. matruchotii, Neisseria*, *Selenomonas noxia,* and *Selenomonas artemidis*. A full list of indicator taxa and related statistics is available in Additional file 7*.*

### Quantitative PCR confirms periodontal disease indicators

There were no significant alterations in bacterial 16S rRNA gene copy numbers (per ng of DNA) in healthy versus diseased plaque (Figure 7). Using species-specific primers, qPCR was used to determine the relative abundance of three species (that is, *P. gingivalis*, *F. alocis*, and *T. forsythia*) in SubG, SupG, and tongue sites using healthy and diseased plaque. Of these species, *P. gingivalis* and *T. forsythia* were validated as the most significantly abundant taxa in the CP group (Figure 7), in agreement with the sequence data. In particular, levels of *P. gingivalis* were elevated in all three sites affected by CP, suggesting that SupG and tongue are as effective as SubG sampling sites for quantification of *P. gingivalis* (Figure 7). The relative number of *T. forsythia* was significantly elevated in diseased samples in SupG and SubG sites, but not in the tongue. The relative abundance of *F. alocis* was elevated significantly in SupG diseased samples, relative to controls. Although its abundance was markedly increased in diseased SubG samples, this result could not be validated on the basis of statistical analyses performed in this study. Some disagreement between the high-throughput sequencing and qPCR was seen. The sequencing results showed a significant increase in *F. alocis* in the disease state in all three sites, but the qPCR results suggested an increase in *F. alocis* in the healthy samples of the tongue site (qPCR data shown in Figure 7, Additional file 5 visualizes the differences between species, and Additional file 6 lists species with significantly different abundances and relevant statistics). An increase in *P. gingivalis* was indicated in all three sites by the qPCR method, but this increase could not be validated with statistical analyses of the sequence data. A significant increase in *T. forsythia* was reported in the SubG and SupG sites by qPCR, but sequencing results identified an increase in this organism in the SubG and tongue sites.

## Discussion

In this study, microbial communities associated with CP and periodontal health were characterized by examining plaque bacteria from SupG, SubG, and tongue sites, providing a comprehensive cross-sectional description of microbiota in the presence of CP and periodontal health. Findings reported here show that most OTUs are shared between periodontal health and disease. Nonetheless, a relatively small proportion of OTUs were distinct to disease and included *T. denticola*, *T. forsythia*, *P. endodontalis*, *Synergistes* OT 362, and *Synergistes* OT 363. Because these disease indicators were present in high abundance in tongue plaque of individuals suffering from CP, we propose that tongue plaque may be used as an easily accessible, non-invasive source to collect plaque to quantify bacterial biomarkers to monitor treatment outcomes against CP over time. In developing such methodologies, it will be useful to evaluate the ratio of selected periodontal disease-associated taxa to health-associated taxa and with larger numbers of human participants for validation. It will also be critically important to differentiate between active and inactive periodontal disease because the microbial communities under these two conditions can vary.

High-throughput sequencing studies conducted to date have examined plaque derived from SubG and SupG sites to differentiate between healthy and periodontal disease consortia [25,27,30]. SubG samples can be more difficult to obtain as compared to samples taken from SupG or tongue sites. Collecting plaque from SubG locations generally requires that dentally trained investigators do most of the sampling. However, if sampling of oral microbes associated with periodontitis were to be done on a larger basis and for large population samples (for example, investigations that seek to correlate periodontitis with non-oral diseases), it would be most advantageous to develop a simpler approach to microbial sampling. Here, we investigated the use of SupG and tongue samples as potential surrogate sites for bacterial biomarker detection under health and CP. The keratinized mucosal surface present in the tongue dorsum is a distinct colonization surface relative to that of tooth structures, which are comprised of highly mineralized tissues such as enamel and cementum. Tooth surfaces are colonized by different consortia depending on the distinct anatomical site and gingival surfaces [31,32]. Not surprisingly, in our ordination plots using SupG, SubG, and tongue plaque, tongue samples clearly separated from tooth plaque samples (Figures 2 and 3A). This separation indicated that bacterial communities present in tongue sites were distinct from those found in SupG and SubG sites. Previously, Griffen and colleagues reported clustering based on disease, although only SubG plaque was sampled in their study [25]. Similar clustering was not seen in our PCoA ordinations (Figure 3). Similar to the weak separation found in our findings, a recent metagenomic and metatranscriptomic study of SubG and SupG plaque failed to find a strong separation between health and CP [33]. The shift from health to disease may be due to a subtle ecological shift and as a result will not appear in the primary axes of the ordination plots. An MRPP analysis, which calculates group separation based on higher dimensional data, would be able to identify a more subtle separation by utilizing all dimensions of the data. MRPP revealed small but significant differences in the community composition between health and diseased states in the combined, SubG, and tongue sites (*P* < 0.05; full MRPP statistics available in Additional file 3).

Previous work demonstrated that certain bacterial complexes associate with each other and potential periodontal diseases [22]. DNA-DNA hybridization was used to monitor 40 bacterial species using 13,261 plaque samples from 185 healthy or periodontal disease patients. By examining pre- and post-therapy bacterial profiles, species prevalence, community ordination, and association with 0 to 3 mm, 4 to 6 mm, and >6 mm probing depths, disease-associated bacteria included *P. gingivalis*, *T. forsythia*, and *T. denticola,* which were highly prevalent in deeper sites that bled upon probing [34]. Prior to this, it was reported that that the probability of finding oral treponemes at sites that harbored *P. gingivalis* was escalated as periodontal destruction was increased [35]. Others demonstrated that species present in disease were also present in the healthy state, albeit in lower proportions [25], while another group demonstrated that SupG sites harbored the same species as SubG sites below the gingival margin, again in smaller proportions [36]. The latter study postulated that this may be conducive to re-infection and individuals with ‘healthy’ sites with abundant disease-indicator organisms may be at higher risk for disease progression. As a result, it may not be unexpected that the Bray-Curtis and UniFrac distance ordinations did not show consistent group separation. Furthermore, additional statistical tests of the datasets may be more informative than ordinations for identifying disease-associated taxa.

In addition to classic periodontitis-associated pathogens mentioned above, we and others detected *F. alocis* in patients with increased periodontal destruction (Figure 6) [37-39]. In this study, *F. alocis* was detected in significantly higher abundances in disease in all three sites (differences between health and disease are visualized in Additional file 5, and statistics are available in Additional file 6). *F. alocis* is a rod-shaped Gram-positive facultative anaerobe that possesses trypsin proteases, invades human cells, resists oxidative stress, and forms biofilms, which are properties conducive to disease initiation and progression [38,40,41]. In particular, the prevalence of *F. alocis* was elevated in patients suffering from generalized aggressive periodontitis and CP, whereas it was rarely detected in the control group resistant to periodontitis [37]. Although this pathogen was shown to colonize apical parts of the pocket in close proximity to the soft tissues, it was also shown to contribute to the structural integrity of these multispecies biofilms. Hence, *F. alocis* was suggested to be a good diagnostic marker organism for periodontal disease [37]. Based on previous investigations, as well as the data shown here, here, we propose that *F. alocis* be included as a novel member in Socransky’s ‘Red complex’ of periodontal pathogens [22].

Comparisons within sites using the qPCR assay demonstrated an increase in bacterial abundance only in the tongue samples derived from patients with CP (Figure 7).The total bacterial abundance was not significantly altered when SubG and SupG sites were compared. This result is in contrast to recent studies that indicated a marked increase in the total bacterial abundance in SubG sites of subjects affected by periodontal disease [36,42]. Our qPCR analysis, as well as those conducted by others, does not account for the total bacterial biomass of plaque samples. As in other studies, the total bacterial load between health and disease states were calculated by normalizing qPCR results with the total nanogram of genomic DNA in each sample. Hence, it is not possible to make inferences about the total bacterial load between health and disease samples (that is, per mg plaque dry weight) using the methods employed in this study. Further, although qPCR was useful for validating our Illumina-based indicator species and results generally agreed, observed trends could not always be statistically validated by both methods. In the case of *F. alocis* in the tongue site, the two methods disagreed. Importantly, differences between the qPCR assay and the high-throughput sequence data methods may have been the cause of discrepancies. The qPCR assay depends on primers that may not be entirely specific to the intended organism. The high-throughput sequence data, on the other hand, is affected by the reference dataset used to classify the sequences. Misclassifications or OTUs not having the required taxonomic resolution would cause discrepancies when compared with qPCR data.

Marked differences in α-diversity between health and disease consortia were not detected (Figure 1). These results are not in agreement with findings reported in two recent high-throughput sequencing studies that reported significant elevations in community α-diversity under health and advanced periodontal disease [25,27]. It is likely that discrepancies in the sampling technique (i.e., curette versus paper-point methods), sampling location, and/or methods of DNA extraction can explain differences in α-diversity measures between this research and previous studies. Other issues that might have also influenced the conclusions could be related to disease activity in either the study reported here or others. In this regard, even if periodontal pockets are identified, this does not mean *a priori* that the disease is in an active state. In such cases, it is also possible that the microbial communities found in patients with active versus inactive periodontitis might actually be different from one another. Hence, further and more detailed study is probably needed to consider active versus inactive disease when taking microbiological samples. Indeed, it might even be possible that a particular shift in the microbiome could be used as a predictive marker for the development of active disease or for the presence of inactive disease. For a better understanding of α-diversity differences, future studies should also consider sampling both periodontal pockets and healthy tissue from the same patient, controlling for the between-patient variability observed here.

## Conclusions

For the first time, we demonstrate that the tongue dorsum and SupG sites can be used as alternative sampling sites for detection and enumeration of bacterial biomarkers associated with CP. Use of plaque from these sites, as opposed to sampling periodontal pockets has important clinical implications. The tongue is an easily accessible body site and sampling the tongue dorsum for plaque collection is convenient and less invasive. This means that others with an interest in examining, for example, correlations between CP and certain non-oral disease (for example, respiratory disease) could conceivably perform meaningful microbiological analyses even if they have no training in the dental clinical sciences. It is also noteworthy that even within dental research settings, ongoing or post-treatment assessments of the microbial population could be carried out by almost any technologist thus reducing the costs of investigation since dentists or dental hygienists would not necessarily be needed for the procedure. Thus, the spectrum of those studying oral health and general health correlations and interactions could be widened considerably, at least where microbiological parameters are needed. Currently, 27 CP indicator organisms identified in this study are being quantified in periodontal and case-matched control samples via qPCR to identify the strongest disease biomarkers for both SupG and tongue sites.

The findings reported here add to knowledge of oral microbial communities associated with periodontitis. Of classic and novel periodontal pathogens identified to date, the role of *F. alocis* as a diagnostic marker for CP is noteworthy. Based on results from this study, as well as data presented by others, we propose that *F. alocis* be included as a novel member in Socransky’s Red complex of periodontal pathogens [22]. Because chronic periodontal destruction was attributed predominantly to proliferation of Gram-negative pathogens, inclusion of *F. alocis* in the Red cluster also changes this classic perspective.

## Methods

For this cross-sectional study, 24 patients were recruited through the University of Toronto Graduate Periodontology and Undergraduate Clinics. The study was approved by the University of Toronto Research Ethics Board (REB 23872), and informed consent to participate in the study was obtained from all patients. Eleven healthy and 13 patients with advanced CP (confirmed by clinical and radiographic examinations) consented to participate in this investigation (Table 1). Patients with systemic disease and/or those who had used antibiotics or had undergone a professional dental cleaning within the last six months were excluded from the study. Both SupG and SubG plaque samples were collected from interproximal sites between teeth 16/15 and 15/14 as well as 26/25 and 25/24 using separate metal curettes. After the SupG plaque was collected, the tooth was wiped with sterile gauze and the subgingival environment was accessed through the periodontal pocket. Samples from each site were placed in phosphate-buffered saline (PBS) and then subsequently pooled into SubG and SupG for each patient; samples were not pooled across patients. Plaque from the tongue was collected using a curette by making two midline scrapes of the dorsal surface. Plaque was then frozen at -80°C until DNA analysis could be carried out. All plaque samples used in this study were collected by the same clinician to limit the variability during plaque sampling. DNA was extracted using the BacReady DNA extraction kit utilizing a single enzyme lysis system as recommended by the supplier (GeneScript USA Inc., Piscataway, NJ, USA). Briefly, 1 μL of the plaque mixture was added to 20 μL of the Buffer BR-A (Genscript, NJ), mixed thoroughly by pipetting and incubated for 10 min at room temperature. These reactions containing template DNA from lysed cells were used for PCR as described below. To validate the use of BacReady DNA kit for DNA extraction, we employed a mock bacterial community that comprised equal numbers of cells of five Gram-negative and Gram-positive species that are found in the oral cavity. These included *Actinomycetes actinomycetemcomitans*, *P. gingivalis*, *T. denticola*, *T. forsythia*, and *Streptococcus mutans*. Lysis mixtures were used for PCR amplification using species-specific primers (not shown), whereas genomic DNA obtained from pure cultures was used as positive controls. Using agarose gel-electrophoresis, we obtained the expected amplicons for all five species (data not shown) using the BacReady kit for cell lysis, which was used to justify our DNA extraction protocol.

A 16S rRNA gene library of approximately 200-bp V3 16S rRNA genes was generated using modified bacterial 341F and 528R primers [43]. Three PCR amplifications were performed for each sample using 50-μl reaction volumes in order to minimize potential amplification bias [44]. Each reaction mixture contained 25 pmol of each primer, 200 μM of each dNTP, 1.5 nM MgCl_2_ and 1 U of Phusion *Taq* polymerase (New England Biolabs, Ipswich, MA, USA), and 10 ng of template DNA. Amplifications were carried out as follows: denaturation at 95°C for 5 min, 20 cycles at 95°C for 1 min, 50°C for 1 min, and 72°C for 1 min, followed by an extension step at 72°C for 7 min. A 2% agarose gel was used to separate products from primers; the correct band was recovered using a QIAquick Gel Extraction Kit as recommended by the manufacturer (Qiagen, Valencia, CA, USA). After measuring sample concentration using a NanoPhotometer (Implen, Denmark), 10 ng/μl aliquots of PCR amplicons were combined prior to paired-end sequencing with individually indexed samples [43]. Sequencing was performed on an Illumina MiSeq (2 × 151 bp using a 300 cycle reagent kit). All sequences were deposited into the European Nucleotide Archive under the project accession number PRJEB6047.

Sequence data were analyzed in four parallel AXIOME (v1.6) analyses (i.e., SubG samples, SupG samples, tongue samples, and all three sites combined). All analyses were conducted using the same configurations. Paired-end reads were assembled using PANDAseq [45], then assembled sequences were clustered into operational taxonomic units (OTUs) at 97% identity using cd-hit-est (v4.5.4) [46]. Multiple sequence alignment of representative sequences was completed with PyNAST (v1.2) via QIIME (v1.6) and FastTreeMP (v2.1.3), with default parameters, to generate a phylogenetic tree of OTUs to calculate UniFrac distances [47-50]. Classification was completed using RDP (v2.2) via QIIME with a confidence cutoff of 0.8 [48,51]. Classifications were rank-flexible, meaning each OTU was only classified down to the lowest taxonomic rank that had 80% or greater posterior probability. A merged Greengenes (October 2012 revision) [52,53] and OSU CORE oral database (9 February 2012 revision) [54] was used for classification. The final OTU table was generated by QIIME. UniFrac-based principal coordinates analysis (PCoA) which was performed through QIIME. Indicator species analysis [29], Bray-Curtis PCoA ordinations, and multiresponse permutation procedure (MRPP) analyses [55], based on nonmetric multidimensional scaling (NMDS) plots using Bray-Curtis distances, were conducted by the AXIOME pipeline [40]. Alpha-diversity plots were generated by Phyloseq (v1.3.14) [56].

### Validation of CP indicator species using quantitative Real-Time PCR (qPCR)

Forty nanograms of DNA from each plaque sample were amplified using qPCR with species-specific primer-pairs for *Tanerella forsythia* (*Tf*: 5′-GGATTGACCACCGGCGAAGACA-3′ and 5′-CGGACACGACGGTTACTCAAATGG-3′)*, P. gingivalis* (*Pg*: 5′-CTTGACTTCAGTGGCGGCAG-3′ and 5′-AGGGAAGACGGTTTTCACCA-3′), and *Filifactor alocis* (*Fa:* 5′-TCGGTGCCGAAGTTAACACA-3′ and 5′-GGGTCCCCGTCAATTCCTTT-3′) as well as universal primers (5′-TCCTACGGGAGGCAGCAGT-3′ and 5′-GGACTACCAGGGTATCTAATCCTGTT-3′), to quantify total bacteria. Each reaction was run in duplicate using the Stratagene Mx3000P cycler (Agilent Technologies, Santa Clara, CA, USA), and reciprocal values of the mean normalized Ct values (Ct_indicator species_/Ct_total bacteria_) were obtained. Significant differences between healthy cohorts and those with CP within each of the respective locations in the oral cavity were calculated based on use of the non-parametric Mann-Whitney test with GraphPad Prism 6 Software (La Jolla, CA, USA) and outliers removed using Grubbs’ Outlier Test (extreme studentized deviate).

## Abbreviations

*CAL*: clinical attachment loss; *CP*: chronic periodontitis; *MRPP*: multiresponse permutation procedure; *NMDS*: non-metric multidimensional scaling; *OT*: oral taxon; *OTU*: operational taxonomic unit; *PCoA*: principal coordinates analysis; *qPCR*: quantitative polymerase chain reaction; *SubG*: subgingival; *SupG*: supragingival.

## Competing interests

The authors declare that they have no competing interests.

## Authors’ contributions

VG helped in the sample collection, gathering of participant data, manuscript writing, data analysis and interpretation. MWH helped in the manuscript writing, data analysis, and interpretation. NS helped in the qPCR assays, and final approval of manuscript. MDJL helped in the research design, analysis and interpretation of results, manuscript revisions, and final approval of manuscript. MG helped in the sample collection, gathering of participant data, manuscript revisions, and final approval of manuscript. HT helped in the sample collection, gathering of participant data, research design, analysis and interpretation of results, manuscript revisions, and final approval of manuscript. DGC helped in the research design, analysis and interpretation of results, manuscript revisions, and final approval of manuscript. JDN helped in the manuscript writing, data and result analysis and interpretation and research design. DBS helped in the manuscript writing, data and results analysis and interpretation, and research design. All authors read and approved the final manuscript.

## Supplementary Material

Additional file 1 **Sample and Patient Metadata.** This includes sample ID, age, weight, health status, and amount patient smokes and drinks. Click here for file

Additional file 2 **UniFrac Principal Coordinates Analysis (PCoA) ordination.** PCoA ordination of UniFrac distances from a phylogenetic tree created by FastTree. A: All samples (SubG, SupG, and Tongue). B: SubG samples. C: SupG samples. D: Tongue samples. Click here for file

Additional file 3 **Multi-Response Permutation Procedure (MRPP) results of Health and Disease.** MRPP results for each site using Bray-Curtis dissimilarity measures. The *A* statistic is a measure of chance-corrected within-group agreement. The closer this value is to 1, the stronger the agreement within a group. The *T* statistic is a sample-size dependent measure of the separation between the health and disease groups, with more negative *T* meaning a stronger separation between groups. Click here for file

Additional file 4 **Relative abundance of genera between healthy and disease samples for separate SubG, SupG, and Tongue samples.** The top ten most abundant genus level classifications with significant differences between health and disease are shown. Significance was assessed with a Mann–Whitney test (*P* <0.05). Disease abundances are shown in pale red, and health abundances in green. “Uncl.” indicates the group was not classified down to genus, and the lowest level classification available is given. A: SubG samples. B: SupG samples. C: Tongue samples. Click here for file

Additional file 5 **Relative abundance of species between healthy and disease samples.** The top ten most abundant species level classifications with significant differences between health and disease are shown. Significance was assessed with a Mann–Whitney test (*P* < 0.05). Disease abundances are shown in pale red, and health abundances in green. “Uncl.” indicates the group was not classified down to species, and the lowest level classification available is given. A: All samples (SubG, SupG, and Tongue). B: SubG samples. C: SupG samples. D: Tongue samples. Click here for file

Additional file 6 **List of species significantly increased under health or disease.** Spreadsheet file containing significance results comparing relative abundance of taxa under health and disease for SubG, SupG, and Tongue sites. Significance was assessed with a Mann–Whitney test (*P* <0.05). Click here for file

Additional file 7 **Dufrêne-Legendre indicator species analysis.** Spreadsheet file containing the detailed indicator OTU lists for the combined, SubG, SupG, and Tongue analyses. All indicator OTUs with *P* < 0.05 are included. Click here for file

## References

[B1] Cho I Blaser MJ The human microbiome: at the interface of health and disease Nat Rev Genet 2012 13 4 260 270 22411464 10.1038/nrg3182 PMC3418802

[B2] Madupu R Szpakowski S Nelson KE Microbiome in human health and disease Sci Prog 2013 96 Pt 2 153 170 23901633 10.3184/003685013X13683759820813 PMC10365526

[B3] Pflughoeft KJ Versalovic J Human microbiome in health and disease Annu Rev Pathol 2011 7 99 122 21910623 10.1146/annurev-pathol-011811-132421

[B4] Wade WG The oral microbiome in health and disease Pharmacol Res 2013 69 1 137 143 10.1016/j.phrs.2012.11.006 23201354

[B5] Dewhirst FE Chen T Izard J Paster BJ Tanner AC Yu WH Lakshmanan A Wade WG The human oral microbiome J Bacteriol 2010 192 19 5002 5017 10.1128/JB.00542-10 20656903 PMC2944498

[B6] Stearns JC Lynch MD Senadheera DB Tenenbaum HC Goldberg MB Cvitkovitch DG Croitoru K Moreno-Hagelsieb G Neufeld JD Bacterial biogeography of the human digestive tract Sci Rep 2012 1 170 22355685 10.1038/srep00170 PMC3240969

[B7] Marsh PD Dental plaque as a biofilm and a microbial community implications for health and disease BMC Oral Health 2006 6 Suppl 1 S14 10.1186/1472-6831-6-S1-S14 16934115 PMC2147593

[B8] Scannapieco FA Bush RB Paju S Associations between periodontal disease and risk for atherosclerosis, cardiovascular disease, and stroke. A systematic review Ann Periodontol 2003 8 1 38 53 10.1902/annals.2003.8.1.38 14971247

[B9] Shangase SL Mohangi GU Hassam-Essa S Wood NH The association between periodontitis and systemic health: an overview SADJ 2013 68 1 10 12 23951755

[B10] Whiley RA Beighton D Winstanley TG Fraser HY Hardie JM *Streptococcus intermedius, Streptococcus constellatus,* and *Streptococcus anginosus* (the *Streptococcus milleri* group): association with different body sites and clinical infections J Clin Microbiol 1992 30 1 243 244 1734062 10.1128/jcm.30.1.243-244.1992 PMC265033

[B11] Parahitiyawa NB Jin LJ Leung WK Yam WC Samaranayake LP Microbiology of odontogenic bacteremia: beyond endocarditis Clin Microbiol Rev 2009 22 1 46 64 10.1128/CMR.00028-08 19136433 PMC2620633

[B12] Scannapieco FA Mylotte JM Relationships between periodontal disease and bacterial pneumonia J Periodontol 1996 67 10 Suppl 1114 1122 8910830 10.1902/jop.1996.67.10s.1114

[B13] Li X Kolltveit KM Tronstad L Olsen I Systemic diseases caused by oral infection Clin Microbiol Rev 2000 13 4 547 558 10.1128/CMR.13.4.547-558.2000 11023956 PMC88948

[B14] Nibali L Farias BC Vajgel A Tu YK Donos N Tooth loss in aggressive periodontitis: a systematic review J Dent Res 2013 92 10 868 875 10.1177/0022034513501878 23955159

[B15] Tumolo AT Effects of periodontitis J Am Dent Assoc 2013 144 10 1100 24080923 10.14219/jada.archive.2013.0024

[B16] Nonnenmacher C Mutters R de Jacoby LF Microbiological characteristics of subgingival microbiota in adult periodontitis, localized juvenile periodontitis and rapidly progressive periodontitis subjects Clin Microbiol Infect 2001 7 4 213 217 10.1046/j.1469-0691.2001.00210.x 11422244

[B17] Brown LJ Johns BA Wall TP The economics of periodontal diseases Periodontology 2002 29 1 223 234 10.1034/j.1600-0757.2002.290111.x 12102710

[B18] Socransky SS Microbiology of periodontal disease-present status and future considerations J Periodontol 1977 48 9 497 504 10.1902/jop.1977.48.9.497 333085

[B19] Socransky SS Haffajee AD The bacterial etiology of destructive periodontal disease: current concepts J Periodontol 1992 63 4 Suppl 322 331 1573546 10.1902/jop.1992.63.4s.322

[B20] Teles FR Teles RP Uzel NG Song XQ Torresyap G Socransky SS Haffajee AD Early microbial succession in redeveloping dental biofilms in periodontal health and disease J Periodontal Res 2012 47 1 95 104 10.1111/j.1600-0765.2011.01409.x 21895662 PMC3253172

[B21] Teles R Sakellari D Teles F Konstantinidis A Kent R Socransky S Haffajee A Relationships among gingival crevicular fluid biomarkers, clinical parameters of periodontal disease, and the subgingival microbiota J Periodontol 2010 81 1 89 98 10.1902/jop.2009.090397 20059421 PMC2805280

[B22] Socransky SS Haffajee AD Cugini MA Smith C Kent RL Microbial complexes in subgingival plaque J Clin Periodontol 1998 25 2 134 144 10.1111/j.1600-051X.1998.tb02419.x 9495612

[B23] Maiden MF Macuch PJ Murray L Tanner A “Checkerboard” DNA-probe analysis and anaerobic culture of initial periodontal lesions Clin Infect Dis 1997 25 Suppl 2 S230 S232 9310688 10.1086/516231

[B24] Do Nascimento C Sato S Mardegan Issa JP Edson Santos Barbosa R Ferreira De Albuquerque Junior R DNA Checkerboard method for bacterial detection of microbiota from teeth and tongue biofilms. A preliminary study Minerva Stomatol 2008 57 11–12 561 567 19092752

[B25] Griffen AL Beall CJ Campbell JH Firestone ND Kumar PS Yang ZK Podar M Leys EJ Distinct and complex bacterial profiles in human periodontitis and health revealed by 16S pyrosequencing ISME J 2012 6 6 1176 1185 10.1038/ismej.2011.191 22170420 PMC3358035

[B26] Wang YL Liou JD Pan WL Association between maternal periodontal disease and preterm delivery and low birth weight Taiwan J Obstet Gynecol 2013 52 1 71 76 10.1016/j.tjog.2013.01.011 23548222

[B27] Abusleme L Dupuy AK Dutzan N Silva N Burleson JA Strausbaugh LD Gamonal J Diaz PI The subgingival microbiome in health and periodontitis and its relationship with community biomass and inflammation ISME J 2013 7 5 1016 1025 10.1038/ismej.2012.174 23303375 PMC3635234

[B28] Wang J Qi J Zhao H He S Zhang Y Wei S Zhao F Metagenomic sequencing reveals microbiota and its functional potential associated with periodontal disease Sci Rep 2013 3 1843 23673380 10.1038/srep01843 PMC3654486

[B29] Dufrene M Legendre P Species assemblages and indicator species: the need for a flexible assymmetrical approach Ecological Monographs 1997 67 3 345 366

[B30] Liu B Faller LL Klitgord N Mazumdar V Ghodsi M Sommer DD Gibbons TR Treangen TJ Chang YC Li S Stine OC Hastruk H Kasif S Segre D Pop M Salomon A Deep sequencing of the oral microbiome reveals signatures of periodontal disease PLoS ONE 2012 7 6 e37919 10.1371/journal.pone.0037919 22675498 PMC3366996

[B31] Segata N Haake S Mannon P Lemon K Waldron L Gevers D Huttenhower C Izard J Composition of the adult digestive tract bacterial microbiome based on seven mouth surfaces, tonsils, throat and stool samples Genome Biol 2012 13 6 R42 10.1186/gb-2012-13-6-r42 22698087 PMC3446314

[B32] Aas JA Griffen AL Dardis SR Lee AM Olsen I Dewhirst FE Leys EJ Paster BJ Bacteria of dental caries in primary and permanent teeth in children and young adults J Clin Microbiol 2008 46 4 1407 1417 10.1128/JCM.01410-07 18216213 PMC2292933

[B33] Duran-Pinedo AE Chen T Teles R Starr JR Wang X Krishnan K Frias-Lopez J Community-wide transcriptome of the oral microbiome in subjects with and without periodontitis ISME J 2014 8 8 1659 1672 10.1038/ismej.2014.23 24599074 PMC4817619

[B34] Kigure T Saito A Seida K Yamada S Ishihara K Okuda K Distribution of *Porphyromonas gingivalis* and *Treponema denticola* in human subgingival plaque at different periodontal pocket depths examined by immunohistochemical methods J Periodontal Res 1995 30 5 332 341 10.1111/j.1600-0765.1995.tb01284.x 7494175

[B35] Grenier D Demonstration of a bimodal coaggregation reaction between *Porphyromonas gingivalis* and *Treponema denticola* Oral Microbiol Immunol 1992 7 5 280 284 10.1111/j.1399-302X.1992.tb00589.x 1337373

[B36] Ximénez-Fyvie LA Haffajee AD Socransky SS Comparison of the microbiota of supra- and subgingival plaque in health and periodontitis J Clin Periodontol 2000 27 9 648 657 10.1034/j.1600-051x.2000.027009648.x 10983598

[B37] Schlafer S Riep B Griffen A Petrich A Hubner J Berning M Friedmann A Gobel U Moter A Filifactor alocis - involvement in periodontal biofilms BMC Microbiol 2010 10 1 66 10.1186/1471-2180-10-66 20193074 PMC2846919

[B38] Wade WG Has the use of molecular methods for the characterization of the human oral microbiome changed our understanding of the role of bacteria in the pathogenesis of periodontal disease? J Clin Periodontol 2011 38 7 16 21323699 10.1111/j.1600-051X.2010.01679.x

[B39] Kumar PS Leys EJ Bryk JM Martinez FJ Moeschberger ML Griffen AL Changes in periodontal health status are associated with bacterial community shifts as assessed by quantitative 16S cloning and sequencing J Clin Microbiol 2006 44 10 3665 3673 10.1128/JCM.00317-06 17021095 PMC1594761

[B40] D’Elios MM Amedei A Cappon A Del Prete G De Bernard M The neutrophil-activating protein of *Helicobacter pylori* (HP-NAP) as an immune modulating agent FEMS Immunol Med Microbiol 2007 50 2 157 164 10.1111/j.1574-695X.2007.00258.x 17521355

[B41] Aruni AW Roy F Fletcher HM *Filifactor alocis* has virulence attributes that can enhance its persistence under oxidative stress conditions and mediate invasion of epithelial cells by *Porphyromonas gingivalis* Infect Immun 2011 79 10 3872 3886 10.1128/IAI.05631-11 21825062 PMC3187275

[B42] Shibli JA Melo L Ferrari DS Figueiredo LC Faveri M Feres M Composition of supra- and subgingival biofilm of subjects with healthy and diseased implants Clin Oral Implants Res 2008 19 10 975 982 10.1111/j.1600-0501.2008.01566.x 18828812

[B43] Bartram AK Lynch MD Stearns JC Moreno-Hagelsieb G Neufeld JD Generation of multimillion-sequence 16S rRNA gene libraries from complex microbial communities by assembling paired-end illumina reads Appl Environ Microbiol 2011 77 11 3846 3852 10.1128/AEM.02772-10 21460107 PMC3127616

[B44] Kennedy K Hall MW Lynch MDJ Moreno-Hagelsieb G Neufeld JD Evaluating bias for Illumina-based bacterial 16S rRNA gene profiles Appl Environ Microbiol 2014 80 18 5717 5722 10.1128/AEM.01451-14 25002428 PMC4178620

[B45] Masella AP Bartram AK Truszkowski JM Brown DG Neufeld JD PANDAseq: paired-end assembler for illumina sequences BMC Bioinformatics 2012 13 31 10.1186/1471-2105-13-31 22333067 PMC3471323

[B46] Li W Godzik A Cd-hit: a fast program for clustering and comparing large sets of protein or nucleotide sequences Bioinformatics 2006 22 13 1658 1659 10.1093/bioinformatics/btl158 16731699

[B47] Caporaso JG Bittinger K Bushman FD DeSantis TZ Andersen GL Knight R PyNAST: a flexible tool for aligning sequences to a template alignment Bioinformatics 2010 26 2 266 267 10.1093/bioinformatics/btp636 19914921 PMC2804299

[B48] Caporaso JG Kuczynski J Stombaugh J Bittinger K Bushman FD Costello EK Fierer N Pena AG Goodrich JK Gordon JI Huttley GA Kelley ST Knights D Koenig JE Ley RE Lozupone CA McDonald D Muegge BD Pirrung M Reeder J Sevinsky JR Turnbaugh PJ Walters WA Widmann J Yatsunenko T Zaneveld J Knight R QIIME allows analysis of high-throughput community sequencing data Nat Methods 2010 7 5 335 336 10.1038/nmeth.f.303 20383131 PMC3156573

[B49] Price MN Dehal PS Arkin AP FastTree 2–approximately maximum-likelihood trees for large alignments PLoS ONE 2010 5 3 e9490 10.1371/journal.pone.0009490 20224823 PMC2835736

[B50] Lozupone C Knight R UniFrac: a new phylogenetic method for comparing microbial communities Appl Environ Microbiol 2005 71 12 8228 8235 10.1128/AEM.71.12.8228-8235.2005 16332807 PMC1317376

[B51] Wang Q Garrity GM Tiedje JM Cole JR Naive Bayesian classifier for rapid assignment of rRNA sequences into the new bacterial taxonomy Appl Environ Microbiol 2007 73 16 5261 5267 10.1128/AEM.00062-07 17586664 PMC1950982

[B52] DeSantis TZ Hugenholtz P Larsen N Rojas M Brodie EL Keller K Huber T Dalevi D Hu P Andersen GL Greengenes, a chimera-checked 16S rRNA gene database and workbench compatible with ARB Appl Environ Microbiol 2006 72 7 5069 5072 10.1128/AEM.03006-05 16820507 PMC1489311

[B53] McDonald D Price MN Goodrich J Nawrocki EP DeSantis TZ Probst A Andersen GL Knight R Hugenholtz P An improved Greengenes taxonomy with explicit ranks for ecological and evolutionary analyses of bacteria and archaea ISME J 2012 6 3 610 618 10.1038/ismej.2011.139 22134646 PMC3280142

[B54] Griffen AL Beall CJ Firestone ND Gross EL DiFranco JM Hardman JH Vriesendorp B Faust RA Janies DA Leys EJ CORE: A Phylogenetically-curated 16S rDNA database of the core oral microbiome PLoS ONE 2011 6 4 e19051 10.1371/journal.pone.0019051 21544197 PMC3081323

[B55] Mielke PW Jr Meteorological Applications of Permutation Techniques based on Distance Functions Handbook of Statistics, Volume 4 1984 813 830

[B56] McMurdie PJ Holmes S phyloseq: an R package for reproducible interactive analysis and graphics of microbiome census data PLoS ONE 2013 8 4 e61217 10.1371/journal.pone.0061217 23630581 PMC3632530

