# Supplementary figures and images for: Bacterial community composition of chronic periodontitis and novel oral sampling sites for detecting disease indicators
Source: Microbiome. 2014 Aug 26;2:32. doi: 10.1186/2049-2618-2-32 (PMC4164120; doi:10.1186/2049-2618-2-32)

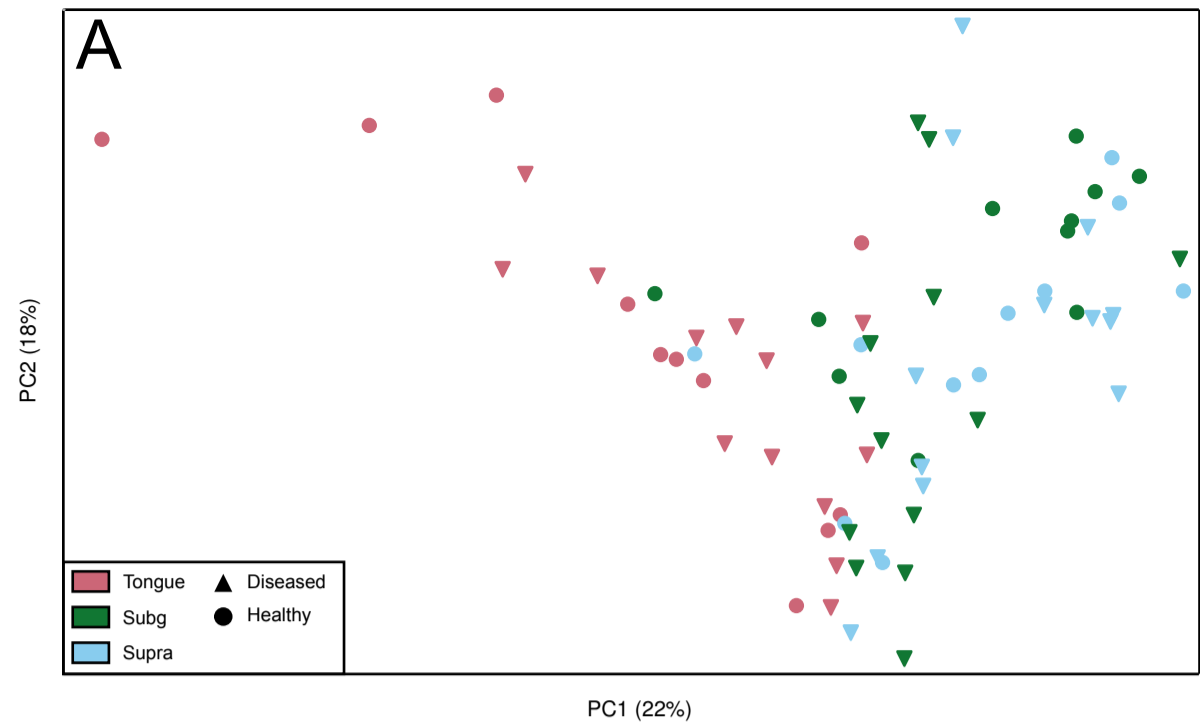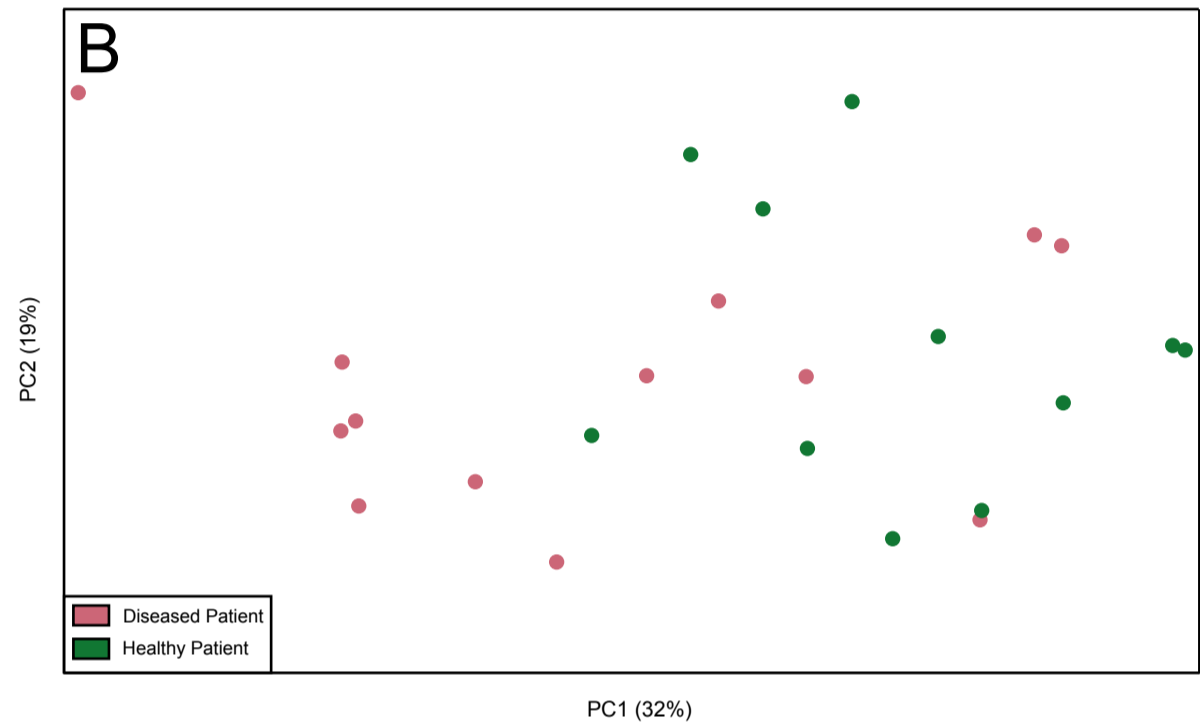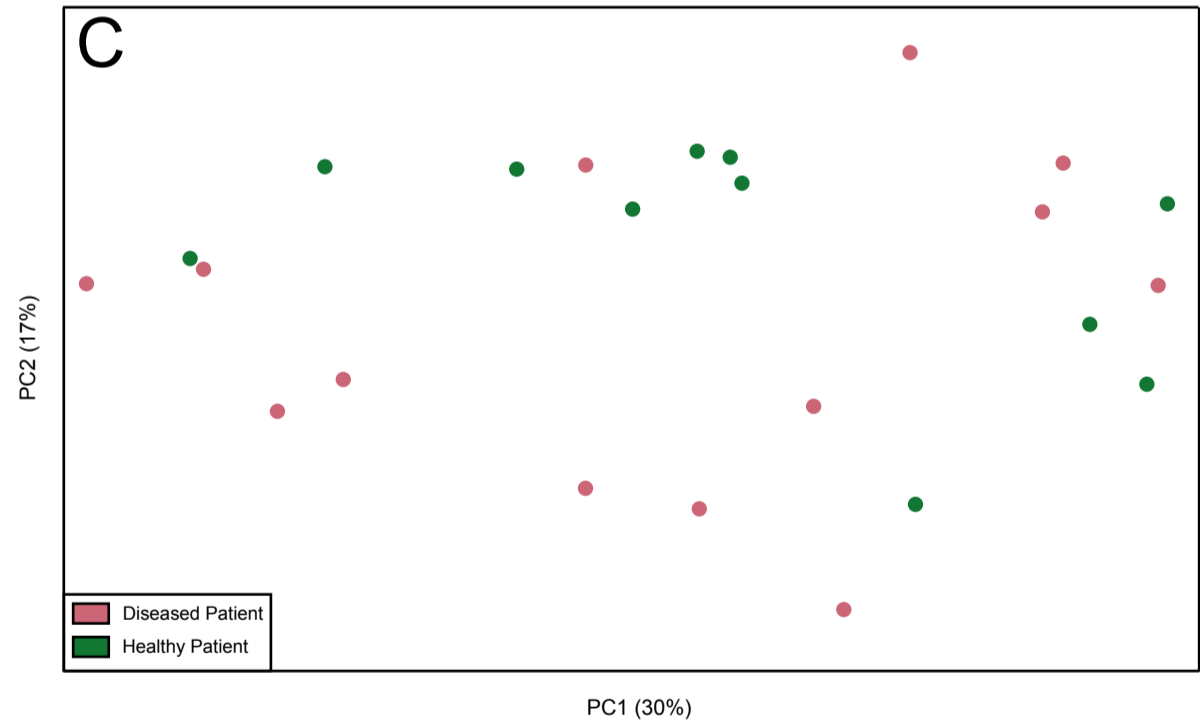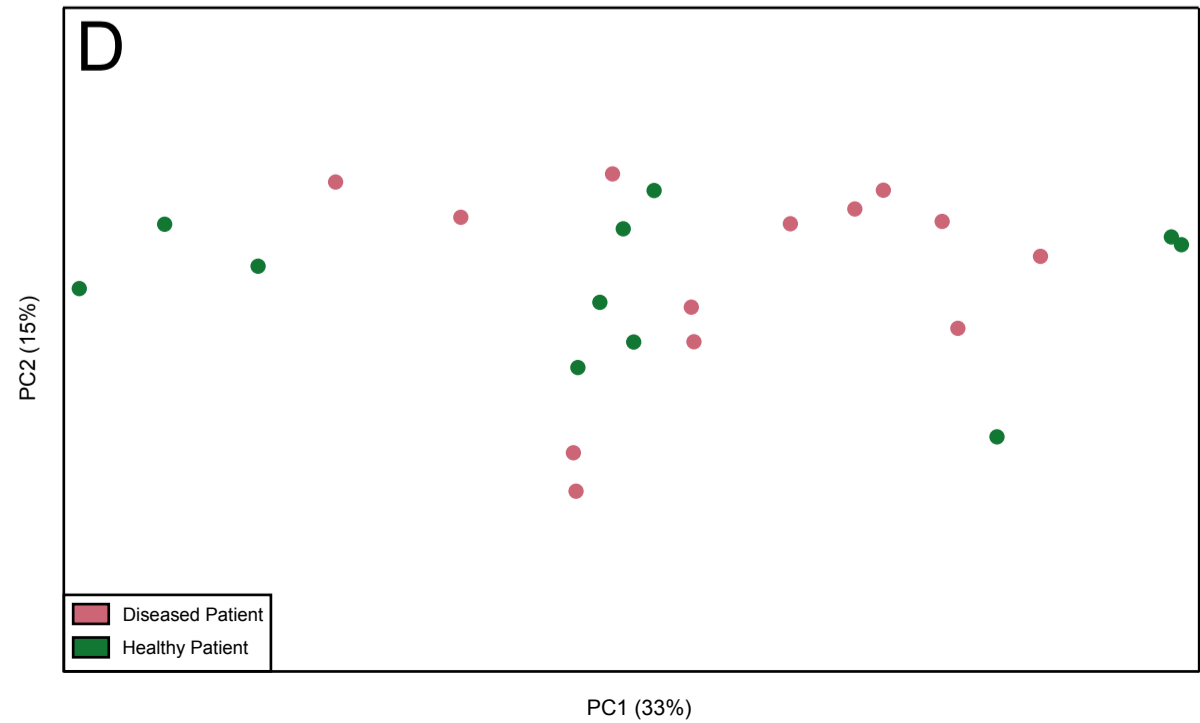

Supplement: Additional file 2 — UniFrac Principal Coordinates Analysis (PCoA) ordination. PCoA ordination of UniFrac distances from a phylogenetic tree created by FastTree. A: All samples (SubG, SupG, and Tongue). B: SubG samples. C: SupG samples. D: Tongue samples. [file 2049-2618-2-32-S2.pdf]

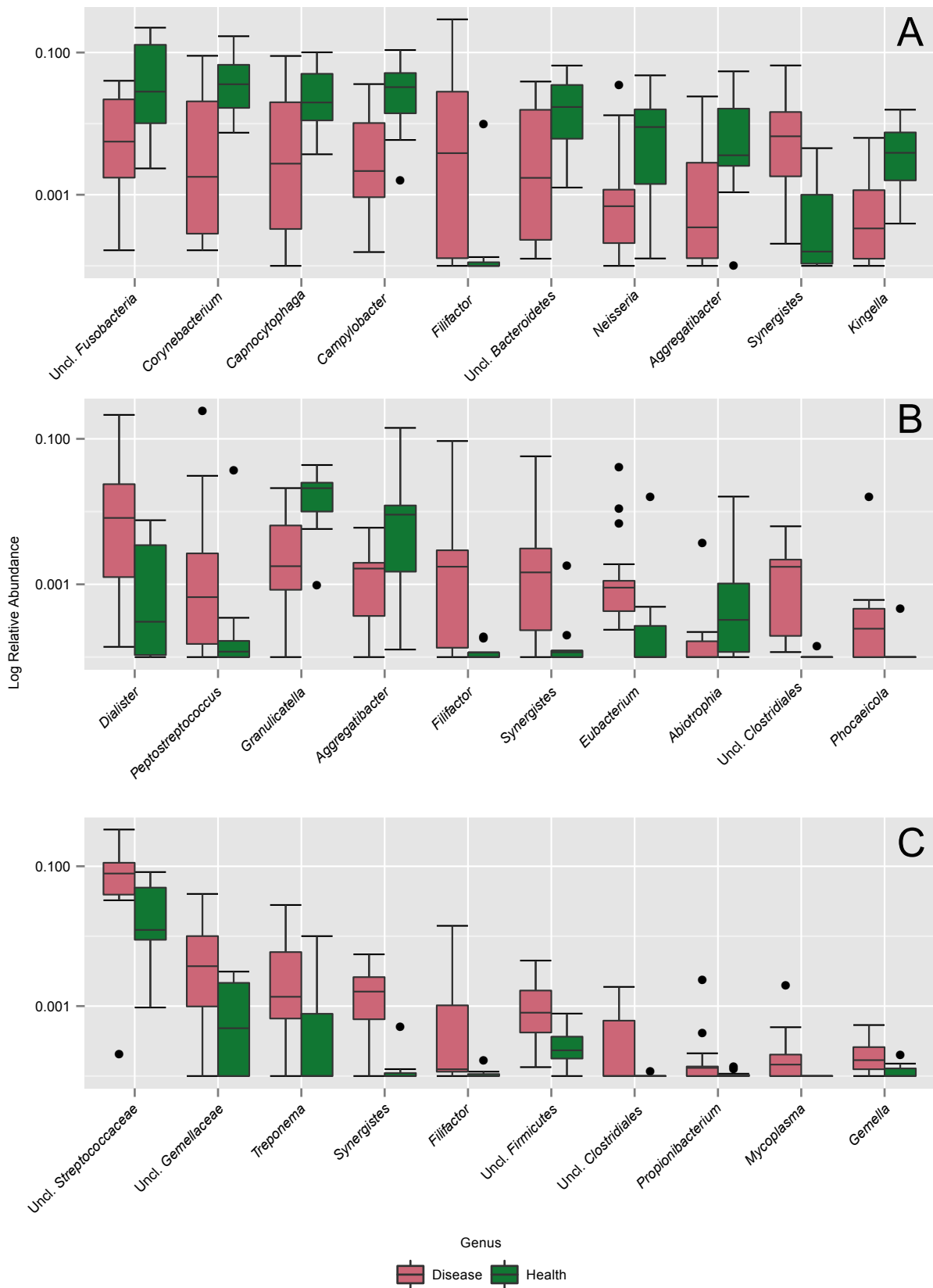

Supplement: Additional file 4 — Relative abundance of genera between healthy and disease samples for separate SubG, SupG, and Tongue samples. The top ten most abundant genus level classifications with significant differences between health and disease are shown. Significance was assessed with a Mann–Whitney test (P <0.05). Disease abundances are shown in pale red, and health abundances in green. “Uncl.” indicates the group was not classified down to genus, and the lowest level classification available is given. A: SubG samples. B: SupG samples. C: Tongue samples. [file 2049-2618-2-32-S4.pdf]

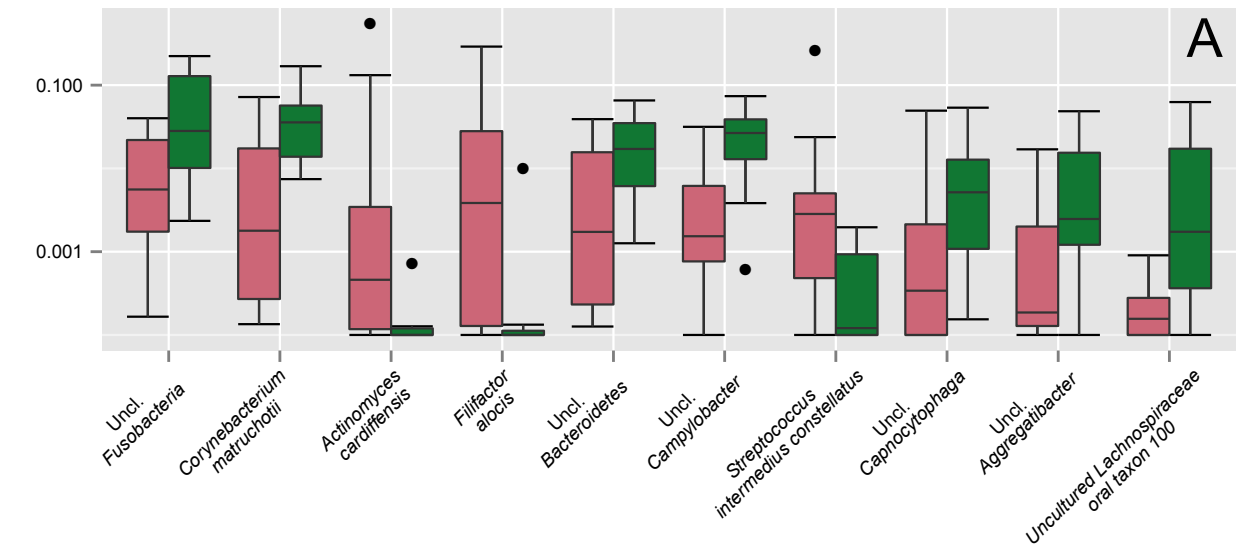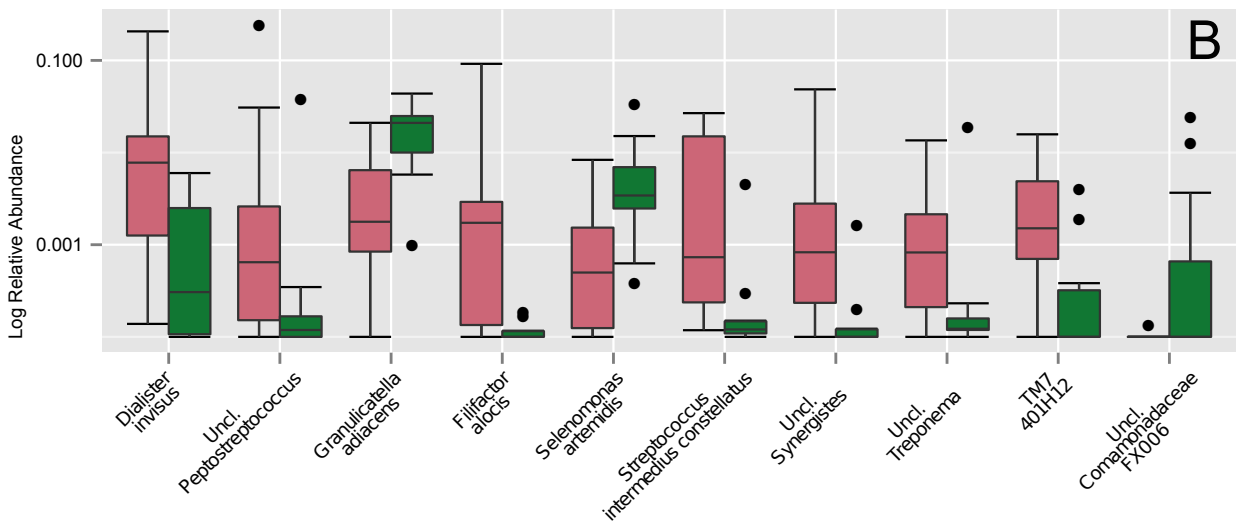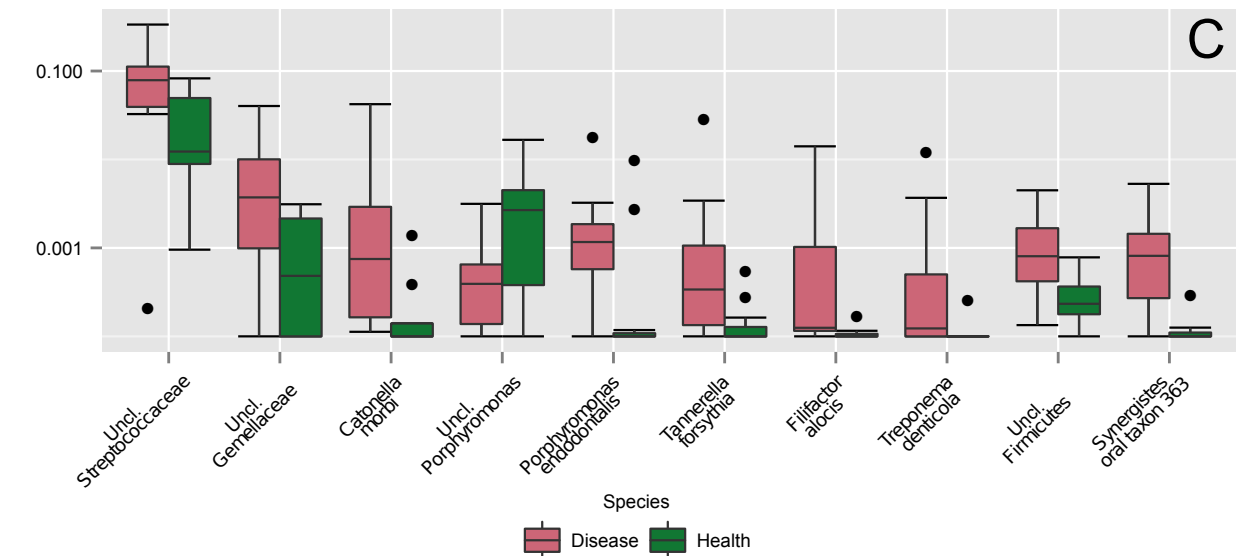

Supplement: Additional file 5 — Relative abundance of species between healthy and disease samples. The top ten most abundant species level classifications with significant differences between health and disease are shown. Significance was assessed with a Mann–Whitney test (P < 0.05). Disease abundances are shown in pale red, and health abundances in green. “Uncl.” indicates the group was not classified down to species, and the lowest level classification available is given. A: All samples (SubG, SupG, and Tongue). B: SubG samples. C: SupG samples. D: Tongue samples. [file 2049-2618-2-32-S5.pdf]
